# Serum metabolic biomarkers distinguish metabolically healthy peripherally obese from unhealthy centrally obese individuals

**DOI:** 10.1186/s12986-016-0095-9

**Published:** 2016-05-12

**Authors:** Xiang Gao, Weidong Zhang, Yongbo Wang, Pardis Pedram, Farrell Cahill, Guangju Zhai, Edward Randell, Wayne Gulliver, Guang Sun

**Affiliations:** College of Food Science and Engineering, Ocean University of China, No.5, Yu Shan Road, Qingdao, Shandong Province China; Faculty of Medicine, Memorial University, 300 Prince Philip Drive, St. John’s, NL Canada; Department of Endocrinology, The First Affiliated Hospital of Dalian Medical University, Dalian, 116000 Liaoning China

**Keywords:** Metabolomics, Healthy peripheral obesity, Unhealthy central obesity, Human, Serum, Biomarkers

## Abstract

**Background:**

Metabolic abnormalities are more associated with central obesity than peripheral obesity, but the underlying mechanisms are largely unknown. The present study was to identify serum metabolic biomarkers which distinguish metabolically unhealthy centrally obese (MUCO) from metabolically healthy peripherally obese (MHPO) individuals.

**Methods:**

A two-stage case–control study design was employed. In the discovery stage, 20 individuals (10 MHPO and 10 MUCO) were included and in the following validation stage, 79 individuals (20 normal weight (NW), 30 MHPO, 29 MUCO) were utilized. Study groups were matched for age, sex, physical activity and total dietary calorie intake with MHPO and MUCO additionally matched for BMI. Metabolic abnormality was defined as: 1) HOMA-IR > 4.27 (90^th^ percentile), 2) high-density lipoprotein cholesterol < 1.03 mmol/L in men and < 1.30 mmol/L in women, 3) fasting blood glucose ≥ 5.6 mmol/L, and 4) waist circumference > 102 cm in men and > 88 cm in women. MUCO individuals had all of these abnormalities whereas MHPO and NW individuals had none of them. A targeted metabolomics approach was performed on fasting serum samples, which can simultaneously identify and quantify 186 metabolites.

**Results:**

In the discovery stage, serum leucine, isoleucine, tyrosine, valine, phenylalanine, alpha-aminoadipic acid, methioninesulfoxide and propionylcarnitine were found to be significantly higher in MUCO, compared with MHPO group after multiple testing adjustment. Significant changes of five metabolites (leucine, isoleucine, valine, alpha-aminoadipic acid, propionylcarnitine) were confirmed in the validation stage.

**Conclusions:**

Significantly higher levels of serum leucine, isoleucine, valine, alpha-aminoadipic acid, propionylcarnitine are characteristic of metabolically unhealthy centrally obese patients. The finding provides novel insights into the pathogenesis of metabolic abnormalities in obesity.

**Electronic supplementary material:**

The online version of this article (doi:10.1186/s12986-016-0095-9) contains supplementary material, which is available to authorized users.

## Background

Over the past 30 years, the prevalence of obesity has been increasing worldwide across all gender and age groups and has reached epidemic proportions in both developed and developing countries [1]. Obesity represents a major public health concern and is associated with increased risk of developing co-morbidities including metabolic syndrome (MS), type 2 diabetes mellitus (T2DM), cardiovascular disease (CVD) and at least a dozen types of cancer [2–5]. Numerous genetic, metabolic and environmental factors alone or more likely in combination lead to the excessive accumulation of body fat, which defines obesity. However, the clinical manifestations of obesity are not homogeneous and accumulating evidence suggests that not all obese individuals necessarily develop metabolic disorders. A sub group of obese people, reported as 6–40 %, are absent of metabolic abnormalities like dyslipidemia, insulin resistance (IR), hypertension and inflammatory profile [6–12], suggesting that a “metabolically healthy obese” phenotype exists. Although, the mechanisms responsible for the existence of metabolically healthy and unhealthy obese phenotypes are not yet clear, body fat distribution is currently the primary candidate due to its crucial role in metabolic health [10, 12]. Visceral fat accumulation or central obesity (apple-shaped individuals) appears to be a more critical factor that linking obesity with the increased risk of developing MS and diabetes than the amount of total body fat or peripheral fat [6, 10–12]. At present, very little is known about the metabolic characteristics of metabolically healthy and unhealthy obesity. Using a more comprehensive screening tool is essential to explore and understand the metabolic profiles of different obesity phenotypes.

Metabolomics is defined as an “omics” technology characteristic of the high-throughput identification and quantification of small molecule (<1500 Da) metabolites in cell, tissue, blood or organism [13]. Previously, metabolomics has been identified as a promising and effective technique to help elucidate the etiology of diseases, such as obesity [14], diabetes [15, 16] and CVDs [17], and assess the effects of natural health products on certain pathological issues [18]. The currently evolving metabolomic techniques brings a wealth of opportunities to seek out, and hopefully develop new biomarkers that may become important tools for identifying diseases, predicting their progression, and determining the effectiveness of therapeutic interventions [19].

To date, only three investigations have implemented metabolomics technology to differentiate the metabolically unhealthy and healthy obese individuals. A study on overweight/obese women with and without MS in Finland found that serum branched-chain amino acids (BCAAs), aromatic amino acids and orosomucoid were associated with all risk factors of MS, with the definition of MS by the presence of any three of the five criteria [20]. Another study in Germany reported that changes of arachidonic acid, glutamine, histidine, spermidine and PC aa C32:3 in cultured human adipocytes distinguish metabolically healthy and unhealthy obese individuals [21]. This study mainly focused on adipocytes and the criteria used to distinguish metabolically healthy from unhealthy obesity was IR alone. A recent study in China found the levels of serum L-kynurenine, glycerophosphocholine, glycerol 1-phosphate, glycolic acid, tagatose, methyl palmitate, and uric acid were significantly different between metabolic healthy (MHO) and abnormal obesity (MAO) [22]. The MAO was defined as having one or more abnormal metabolic indexes, including hyperglycemia, hypertension and dyslipidemia, while MHO had none of them. However, none of these studies systematically defined metabolically healthy or unhealthy obesity nor did they consider the critical importance of body fat distribution.

In the present study, we aimed to find important metabolites that distinguish metabolically healthy peripheral obese (MHPO) from metabolically unhealthy central obese (MUCO) individuals with a more stringent definition along with a significant emphasis placed upon body fat distribution. A targeted metabolomics methodology was applied, which has broadly been used in the study of metabolic diseases [23, 24]. The potential metabolites discovered from this current investigation will more accurately represent the metabolic route discrepancy between "metabolically healthy obesity" and "metabolically unhealthy obesity" than any other studies to date. Moreover, these potential metabolites could lead to the discovery of a number of important biomarkers for central obesity related metabolic abnormalities.

## Methods

### Ethics statement

This study received ethical approval from Health Research Ethics Authority of the Faculty of Medicine of Memorial University, St. John’s, Newfoundland, Canada, [with Project Identification Code #10.33 (latest date of approval: February 11, 2016.)]. Written informed consent was obtained from all of the volunteers.

### Study population

We used a two-stage case–control study design, namely, discovery and validation phases. Individuals for both phases were selected from the ongoing CODING (Complex Diseases in the Newfoundland Population: Environment and Genetics) study [25–29]. Inclusion criteria for the CODING study are: 1) at least a third generation Newfoundlander, 2) between the ages of 20 and 79 years old, 3) not pregnant at the time of study. The metabolic characteristics used for the classification of subjects being of metabolically unhealthy or healthy are as follows:

Metabolically Unhealthy Central Obesity (MUCO) - 1) homeostasis model assessment of insulin resistance (HOMA-IR) > 4.27 (90^th^ percentile) [9, 11], 2) high-density lipoprotein cholesterol (HDL-C) level < 1.03 mmol/L in men and < 1.30 mmol/L in women [9, 11], 3) fasting blood glucose ≥ 5.6 mmol/L [9, 11], and 4) waist circumference > 102 cm in men and > 88 cm in women [9, 11];

Metabolically Healthy Peripheral Obesity (MHPO) - 1) HOMA-IR < 4.27 [9, 11], 2) HDL-C level ≥ 1.03 mmol/L in men and ≥ 1.30 mmol/L in women [9, 11], 3) fasting blood glucose < 5.6 mmol/L [9, 11], and 4) waist circumference ≤ 102 cm in men and ≤ 88 cm in women [9, 11].

In the discovery stage, two groups of obese individuals (10 MUCO and 10 MHPO) were selected. All study participants were classified as obese according to the World Health Organization (WHO) criteria for obesity (body mass index, BMI ≥ 30 kg/m^2^). Subjects of the two groups were matched for age, BMI, total dietary calorie intake, and physical activity level.

In the follow validation stage, a normal weight group (NW, 20 subjects) and two obese groups (29 subjects for MUCO group and 30 subjects for MHPO group) were selected. The metabolic characteristics used to distinguish the two obese groups were the same as during the discovery stage, except BMI was expanded to above 27.2 due to the difficulty in identifying samples meeting the stringent criteria of the studying groups. The metabolic characteristics for NW group were same to MHPO group except with 18 < BMI < 25. Subjects of the three groups were also matched for age, sex, total dietary calorie intake, and physical activity level, with MUCO and MHPO additionally matched for BMI.

### Anthropometric and body composition measurements

All measurements were performed in the morning following a 12-h fasting period. Subjects were weighed (Health O Meter, Bridgeview, IL) to the nearest 0.1 kg in standardized clothing (hospital gown). Height was measured to the nearest 0.1 cm using a fixed stadiometer. BMI was calculated from weight and height in kilograms per square meter. Waist circumference was measured midway between the lowest rib and iliac crest and evaluated using a measuring tape to the nearest 0.1 cm. Blood pressure (BP) was measured twice by manual oscillometric methods in the morning after sitting for 10 minutes after the subjects arrived at the laboratory.

Whole body composition measurements including fat mass, lean body mass were measured using dual-energy X-ray absorptiometry (DXA) Lunar Prodigy (GE Medical Systems, Madison, WI) equipped with enCORE software package (GE Medical Systems) Version 12.3 [11, 27]. The total percent body fat (BF%), percent trunk fat (TF%), and percent android fat (AF%) were determined. The Lunar Prodigy software system determines automatically the regions. Trunk fat region is from the top of the shoulders to the top of the iliac crest, while the android fat region is the top of the second lumbar vertebra to the top of the iliac crest. Visceral adipose tissue content was estimated by CoreScan [30, 31] within the android region and percent visceral fat (VF%) was determined.

### Dietary assessment

Dietary intake patterns of each participant were assessed using Willett Food Frequency Questionnaire (FFQ), a semi-quantitative method for the assessment of dietary intake patterns. The Willett FFQ is the most widely used dietary intake questionnaire for the study of nutritional information at the population level [32–34]. For each food item listed, participants had to indicate their average use of the specified amount per week over the last year. Based on the choice selected, the amount was converted to a mean daily intake value. The daily intake for each food item consumed was entered into a meal plan using NutriBase Clinical Nutrition Manager (version 8.2.0; CyberSoft Inc, Arizona) and the daily macronutrient, micronutrient and total calorie intakes were automatically computed by the NutriBase software [35].

### Physical activity

Physical activity levels were measured using the ARIC-Baecke Questionnaire, which consists of a Work Index, Sports Index, and Leisure Time Activity Index [36]. All responses from this questionnaire were scored based on a five point scale with the exception of the name of the participant’s main occupation and the type of sports played. Three levels of physical activity (low, medium and high) were defined for occupation and sports. Physical activity was then measured via assessment of the number of hours spent doing the activity per week, the number of months spent doing the activity per year and the assigned exertion level. The work, sports, and leisure time activity indices were added together to give an estimate of total physical activity.

### Serum lipids, glucose and insulin measurement

Venous blood samples were obtained from all volunteers in the morning after an overnight fast (12 h). Serum samples were isolated from blood and stored at −80 °C for subsequent analysis. Concentrations of serum high-density lipoprotein cholesterol (HDL-C), triglycerides (TG) and glucose were analyzed using Synchron reagents with an Lx20 analyzer (Beckman Coulter Inc., Fremont, CA, USA). Additionally, the serum insulin level was measured using an immunoassay analyzer (Immulite; DPC, Los Angeles, CA, USA) [11]. HOMA-IR [37] was calculated as follows: HOMA-IR = [(Fasting Insulin (mU/L) × Fasting Glucose (mmol/L))/22.5]

### Serum metabolites measurement

Metabolic profiling was performed by using the Waters XEVO TQ MS system (Waters Limited, Mississauga, Ontario, Canada) coupled with the Biocrates AbsoluteIDQ p180 kit (Innsbruck, Austria), which can simultaneously identify and quantify 186 metabolites including 21 amino acids, 19 biogenic amines, 40 acylcarnitines (including free carnitine), 15 sphingomyelins, 90 glycerophospholipids (14 lysophosphatidylcholines (lysoPC) and 76 phosphatidylcholines (PC)) and 1 hexose (>90 % is glucose). The assay procedures of the kit as well as the metabolite nomenclature have been described in detail previously [23, 24].

### Statistical analyses

Data of the general characteristics of the study participants are presented as means ± SDs. Differences in anthropometry, dietary intakes and physical activity were assessed using Student’s *t*-test. The sex ratio was analyzed by *chi-square* tests. SPSS software version 19.0 (SPSS Inc, Chicago, IL, USA) was used for these analyses. All tests were two-sided and a *p*-value less than 0.05 was considered to be statistically significant.

In the discovery stage, the Partial least squares Discriminant Analysis (PLS-DA) method was used to identify the characteristic metabolites with significant difference between the two groups, using SIMCA-P 11.5 (Umetrics AB, Umea, Sweden) software. Since there were vast differences in the absolute concentrations among different metabolites, all data were mean-centered and standardized before analyses. In PLS-DA, the R2X, R2Y and Q2 (cum) parameters were used for the model evaluation, representing the explanation, fitness and prediction power respectively. R2X is the percentage of all response variables explained by the model. R2Y describes the percentage of variation explained by the model. Q2 shows the predictive value of the model. The importance of each metabolite in the PLS-DA was evaluated by variable importance in the projection (VIP) score. The VIP score positively reflects the metabolite’s influence on the classification, and metabolites with VIP > 1 were considered important in the study. Additionally, the Kruskal-Wallis test was executed using Multi Experiment View (V.4.9) software to determine the significant metabolites. The significance level was defined as *p* < 0.05. Those with VIP > 1 and *p* < 0.05 were recognized as the most important metabolites and Bonferroni method (p_Bonferroni_ = 1–0.95^1/n^) was used to correct for multiple testing in different categories of metabolites. A *p*-value ≤ p_Bonferroni_ was considered to be statistically significant; hexose: ≤ 0.05, amino acid: ≤ 0.005, biogenic amines: ≤ 0.013, acylcarnitines: ≤ 0.004, glycerophospholipids: ≤ 0.006.

In the following validation stage, the normal distributions of the 8 statistically significant metabolites survived from the discovery study were analyzed. Logarithmic transformation was used for the variables that did not show normal distribution. One-Way ANOVA followed by *Tukey* test was used to analyze significant difference between groups by SPSS software version 19.0. Bonferroni method was used to correct for multiple testing. A *p*-value ≤ 0.005 was considered to be statistically significant.

## Results

### Demographic and metabolic characteristics of participants

Twenty obese subjects were included in the discovery stage. The general characteristics of the subjects in the discovery stage are shown in Table 1. There were no significant differences for age, BMI, sex ratio, BF%, TF%, AF%, systolic blood pressure (SBP), diastolic blood pressure (DBP), dietary food intake and physical activity between the two groups. Waist circumference, visceral fat mass, VF%, serum TG, glucose, insulin levels and HOMA-IR value in MUCO group were significantly higher than in MHPO group (*p* < 0.05). HDL-C level was lower in MUCO group than in MHPO group (*p* < 0.001).

**Table 1:** Characteristics of the study participants in the discovery stage

Variables	MUCO	MHPO	*P*-value
Age (years)	49.5 ± 4.8	43.4 ± 11.7	0.154
Sex(F/M)	6/4	8/2	0.628^a^
Anthropometry			
BMI (kg/m^2^)	31.8 ± 1.6	32.2 ± 2.1	0.661
Waist Circumference(cm)	107.6 ± 7.1	88.5 ± 18.2	0.004
BF %	36.4 ± 8.6	42.4 ± 10.8	0.189
TF %	41.7 ± 7.1	45.3 ± 9.5	0.343
AF%	48.8 ± 6.5	51.1 ± 8.6	0.520
Visceral fat (g)	1885.4 ± 369.2	1087.2 ± 663.2	0.004
VF%	5.9 ± 1.6	2.7 ± 1.3	0.000
Metabolic Profile			
SBP (mmHg)	133.9 ± 10.7	127.0 ± 20.3	0.650
DBP (mmHg)	88.6 ± 5.9	85.6 ± 10.8	0.318
TG (mmol/L)	2.4 ± 1.2	1.2 ± 0.4	0.025
HDL-C (mmol/L)	1.0 ± 0.1	1.5 ± 0.2	<0.001
Glucose (mmol/L)	7.1 ± 2.0	4.9 ± 0.3	<0.001
Insulin (pmol/L)	281.9 ± 343.8	54.8 ± 22.1	<0.001
HOMA-IR	14.7 ± 22.3	1.7 ± 0.8	<0.001
Diet and Physical Activity			
Caloric intake (kcal/day)	2057.3 ± 1093.9	1852. 0 ± 846.5	0.662
Protein intake (g/day)	91.0 ± 80.7	72.6 ± 22.5	0.591
FAT intake (g/day)	59.4 ± 29.4	54.5 ± 37.6	0.762
Carbohydrate (g/day)	277.1 ± 160.6	271.2 ± 147.5	0.936
Physical activity level	7.8 ± 1.3	7.7 ± 1.8	0.875

All values are means ± SDs. MUCO: metabolically unhealthy central obesity; MHPO: metabolically healthy peripheral obesity; The student’s *t*-test was set to *p* < 0.05. Logarithmic transformation was used for the variables that did not have normal distribution (Insulin, glucose, HOMA-IR, TG and Protein intake)

^a^Analyzed by *chi-square* tests by SPSS, statistical significance was set to *p* < 0.05

The general characteristics of the subjects in the validation stage are shown in Table 2. There were no significant differences for age, sex ratio, dietary food intakes and physical activity among the three groups. BMI, waist circumference, BF%, TF%, AF%, visceral fat mass were significantly lower in NW group, compared with the two obese groups (*p* < 0.001). VF% was significant higher in MUCO group than NW group (*p* < 0.001), but no significant difference was found between MHPO and NW groups. There were no differences for BMI, BF%, TF% and AF% between the two obese groups, while waist circumference, visceral fat mass and VF% were significantly lower in MHPO group compared to MUCO group (*p* < 0.01). SBP, serum TG, glucose, insulin levels and HOMA-IR value in MUCO group were significantly higher (*p* < 0.05 for all), and serum HDL-C level were significantly lower (*p* < 0.01) than in NW group. DBP, serum insulin level and HOMA-IR value were significantly higher in MHPO group than in NW group (*p* < 0.05 for all), while there were no differences for SBP, serum TG, HDL-C, glucose levels between the two groups. Compared to MUCO group, serum TG, glucose, insulin levels and HOMA-IR value were significantly lower and serum HDL-C level was significantly higher in MHPO group (*p* < 0.001 for all), while there was no difference for blood pressure between the two groups.

**Table 2:** Characteristics of the study participants in the validation stage

Variables	NW(*n* = 20)	MUCO(29)	MHPO (30)	*P1*	*P2*	*P3*
Age (years)	44.9 ± 11.4	49.3 ± 11.4	43.4 ± 11.8	0.384	0.880	0.115
Sex (F/M)	10/10	15/14	15/15	1.000^*a*^	1.000^*a*^	1.000^*a*^
Anthropometry						
BMI (kg/m^2^)	21.7 ± 0.8	31.6 ± 3.2	30.1 ± 1.4	0.000	0.000	0.066
Waist Circumference(cm)	80.7 ± 6.4	107.5 ± 9.5	92.1 ± 7.6	0.000	0.000	0.000
BF %	25.3 ± 8.6	36.5 ± 6.9	35.6 ± 9.2	0.000	0.000	0.924
TF %	26.9 ± 8.6	41.6 ± 5.8	39.8 ± 7.2	0.000	0.000	0.607
AF%	31.9 ± 11.8	48.7 ± 5.7	45.9 ± 6.6	0.000	0.000	0.247
Visceral fat(g)	354.3 ± 295.9	2077.8 ± 814.4	1120.00 ± 641.4	0.000	0.000	0.000
VF%	2.5 ± 2.1	6.5 ± 2.3	4.2 ± 2.8	0.000	0.057	0.002
Metabolic Profile						
SBP (mmHg)	122.4 ± 14.4	137.4 ± 14.3	130.1 ± 15.9	0.003	0.192	0.165
DBP (mmHg)	78.1 ± 9.3	84.3 ± 10.9	85.5 ± 8.3	0.072	0.028	0.890
TG (mmol/L)	0.87 ± 0.29	2.79 ± 1.27	1.11 ± 0.53	0.000	0.060	0.000
HDL-C (mmol/L)	1.6 ± 0.23	0.99 ± 0.17	1.5 ± 0.4	0.000	0.521	0.000
Glucose (mmol/L)	5.0 ± 0.28	7.3 ± 2.8	4.9 ± 0.34	0.000	0.931	0.000
Insulin (pmol/L)	38.9 ± 20.3	153.1 ± 48.6	58.5 ± 32.5	0.000	0.035	0.000
HOMA-IR	1.2 ± 0.6	7.1 ± 3.9	1.9 ± 1.0	0.000	0.029	0.000
Diet and Physical Activity						
Caloric intake (kcal/day)	1896.2 ± 1199.7	1509.3 ± 568.2	1935.6 ± 789.5	0.292	0.999	0.210
Protein intake (g/day)	65.8 ± 36.5	60.6 ± 32.7	73.1 ± 34.5	0.601	0.729	0.152
Fat intake (g/day)	57.0 ± 22.7	50.2 ± 19.6	55.2 ± 20.4	0.800	0.984	0.863
Carbohydrate (g/day)	238.6 ± 128.7	201.9 ± 87.1	248.9 ± 120.4	0.369	1.000	0.074
Physical activity level	8.1 ± 1.7	7.3 ± 1.2	8.1 ± 1.4	0.090	0.974	0.089

All values are means ± SDs. The One-Way ANCOVA followed by *Tukey* test was set to *p* < 0.05. NW: Normal Wight; MUCO: metabolically unhealthy central obesity; MHPO: metabolically healthy peripheral obesity; *P1:*The P value between MUCO and NW groups; *P2:*The P value between MHPO and NW groups; *P3:*The P value between MUCO and MHPO groups; Logarithmic transformation was used for the variables that did not have normal distribution (Insulin, glucose, HOMA-IR, TG)

^a^ Analyzed by *chi-square* tests by SPSS, statistical significance was set to *p* < 0.05

### Identified metabolites in participant’s serum

Over 95 % of the metabolites (178/186) were successfully determined in each sample. These included 40 acylcarnitines (including free carnitine), 21 amino acids, 12 biogenic amines, 89 glycerophospholipids (14 lysoPC, 75 PC), 15 sphingomyelins and hexose (>90 % is glucose), as shown in Additional file 1: Table S1.

### Metabolomics profiles changes in MHPO and MUCO groups in the discovery stage

**Fig. 1:**
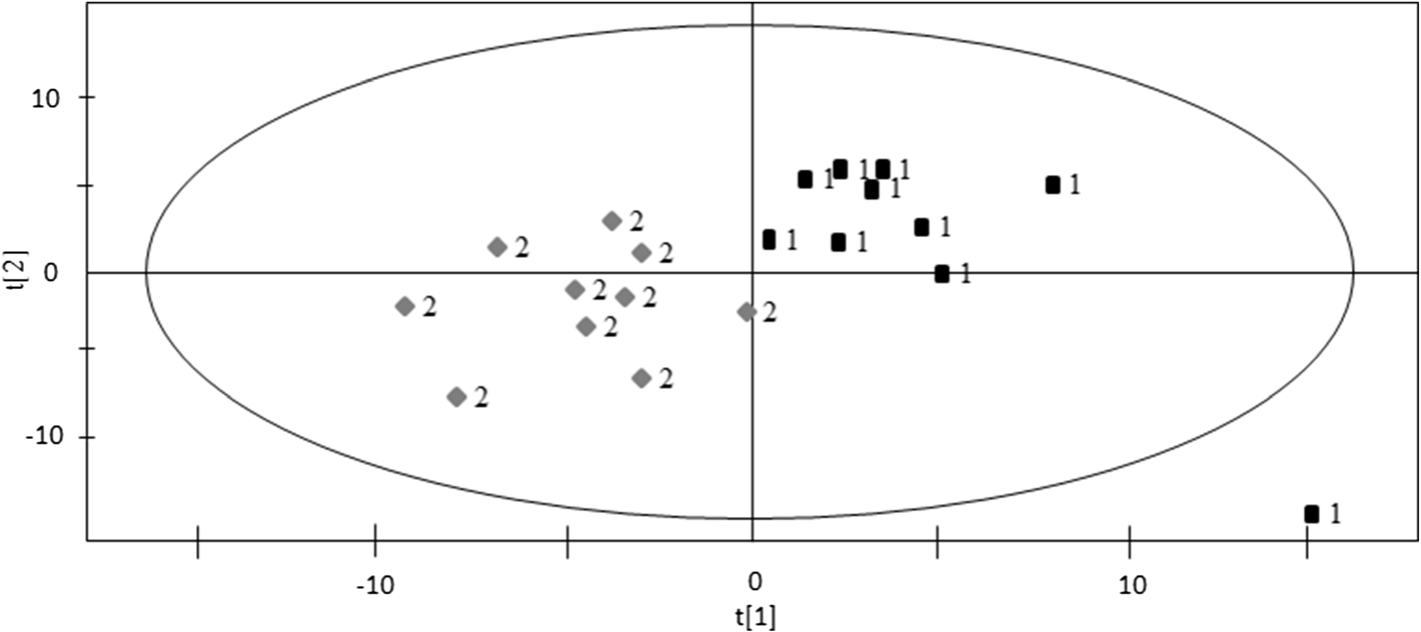
PLS-DA score plots of MUCO and MHPO groups. “1” represent metabolically healthy peripheral obesity (MHPO) group; “2” represent metabolically unhealthy central obesity (MUCO) group

PLS-DA results were presented in Fig. 1. In the constructed PLS-DA model, R2X = 0.422, R2Y = 0.801 and a good prediction parameter Q2 (cum) =0.571. The metabolites with the VIP > 1 were regarded as important in the classification of the two groups. The significant metabolites were further evaluated by the Kruskal-Wallis test with a threshold of *p* < 0.05. The results of metabolites with VIP > 1 and *p* < 0.05 are shown in Table 3.

**Table 3:** The variable importance in the projection (VIP) values^*a*^ and p values of identified metabolites between MHPO and MUCO groups in the discovery stage (VIP > 1, *p*-value < 0.05)

Metabolites	VIP value	*P*-value	Metabolites	VIP value	*P*-value
Hexoses	1.87725	0.000157^*^	C4:1	1.55489	0.010843
Tyrosine	1.95689	0.000507^*^	C10:2	1.55734	0.012379
Leucine	2.00158	0.000669^*^	C6(C4:1-DC)	1.16246	0.015448
Isoleucine	1.91919	0.000881^*^	C0	1.26561	0.015564
Valine	1.93636	0.000881^*^	C6:1	1.36558	0.018482
Phenylalanine	1.78606	0.003197^*^	C16-OH	1.61161	0.024043
Lysine	1.51144	0.008151	C5-DC(C6-OH)	1.30387	0.027184
Histidine	1.55651	0.015564	C16	1.20206	0.033822
Methionine	1.55505	0.015526	C10:1	1.2771	0.041174
Alanine	1.47744	0.019110	C3-DC(C4-OH)	1.0104	0.049025
Proline	1.37059	0.023342	PC aa C40:2	1.30675	0.041250
Glutamine	1.42081	0.041250	PC ae C38:4	1.37446	0.019110
alphaAAA	1.72871	0.001152^*^	PC ae C342	1.11563	0.025692
MetSO	1.57108	0.007564^*^	PC ae C36:4	1.18682	0.028366
Kynurenine	1.41756	0.028366	PC ae C38:5	1.25626	0.034294
Sarcosine	1.3291	0.028366	PC ae C42:0	1.13183	0.041250
C3	1.53624	0.003299^*^	PC ae C36:3	1.15062	0.049366
C4	1.45988	0.006502	PC ae C38:6	1.15541	0.049366
C5:1	1.45929	0.008849	lysoPC a C28:1	1.22246	0.049366
C5	1.44608	0.009056			

^*a*^Higher VIP values indicate a stronger influence of the metabolite in distinguishing different groups. Kruskal-Wallis test set to *p* < 0.05. *MUCO* metabolically unhealthy central obesity; *MHPO* metabolically healthy peripheral obesity; Hexose (mainly glucose); alphaAAA, alpha-Aminoadipic acid; Met-SO, Methioninesulfoxide. Asterisk (*) marks statistical significance after Bonferroni correction

Compared with MHPO group, 39 metabolites were successfully identified in MUCO group. 11 amino acids (alanine, glutamine, histidine, isoleucine, leucine, lysine, methionine, phenylalanine, proline, tyrosine, valine), 4 biogenic amines (alphaAAA, Met-SO, kynurenine, sarcosine), free carnitine (C0) plus 13 acylcarnitines (C3, C3DC(C4OH), C4, C4:1, C5, C5:1, C5-DC(C6-OH), C6(C4:1-DC), C6:1, C10:1, C10:2, C16, C16OH), and hexose (>90 % is glucose) were significantly higher and 9 glycerophospholipids (lysoPC C28:1, PC aa C40:2, PC ae C34:2/C36:3/C36:4/C38:4/C38:5/C38:6/C42:0) were significantly lower in MUCO group.

While, after multiple testing adjustment, hexose, leucine, isoleucine, tyrosine, valine, phenylalanine, alphaAAA, Met-SO and C3 were still statistically significantly different between the two groups. However, glucose accounted for 90 % of the hexose in the metabolomics approach used, thus the elevation of hexose in the MUCO subjects is probably owing to the high level of fasting glucose. Therefore, all these metabolites except hexose were identified as the key metabolites distinguishing MUCO and MHPO groups and were further examined in the validation stage.

### Metabolomics profiles changes among groups in the validation stage

The significant differences of 8 metabolites were further examined during the validation stage. Five metabolites were identified as statistically significant among the three groups. As shown in Table 4, serum leucine, isoleucine, valine, alphaAAA, C3 levels were significant higher in MUCO group compared to NM or MHPO groups after multiple testing adjustment (*p* < 0.001). There were no statistically significant differences on the concentration of the 5 metabolites between MHPO and NW groups.

**Table 4:** Metabolites with significant difference among groups during the validation stage

Metabolites (umol/L)	NM	MUCO	MHPO	*P1*	*P2*	*P3*
Leucine	189.5 ± 37.6	321.5 ± 36.8	216.6 ± 32.6	0.000	0.346	0.000
Isoleucine	87.7 ± 48.2	132.0 ± 52.7	92.5 ± 45.2	0.000	0.584	0.000
Valine	297.8 ± 50.1	388.5 ± 53.4	291.3 ± 43.4	0.000	0.917	0.000
alphaAAA	2.45 ± 0.24	3.10 ± 0.40	2.62 ± 0.29	0.000	0.138	0.000
C3	0.30 ± 0.08	0.42 ± 0.11	0.29 ± 0.11	0.000	0.976	0.000

All values are means ± SDs. The One-Way ANOVA followed by *Tukey* test was set to *p* < 0.05. *NW* normal weight, *MUCO* metabolically unhealthy central obesity, *MHPO* metabolically healthy peripheral obesity; alphaAAA, alpha-aminoadipic acid. Logarithmic transformation was used for the variables that did not have normal distribution (alphaAAA); *P1:* The P value between MUCO and NW groups; *P2:* The P value between MHPO and NW groups; *P3:*The P value between MUPO and MHPO groups

## Discussion

To the best of our knowledge, this study is the first to identify serum metabolic biomarkers in MUCO focusing on fat distribution factors and using a metabolomics technology. We discovered significant increases in 5 serum metabolites (leucine, isoleucine, valine, alphaAAA and C3 acylcarnitine) that distinguished metabolically unhealthy centrally obese from metabolically healthy peripherally obese patients.

Obesity is one of the primary risk factor for diabetes and other metabolic conditions [2]. However, paradoxically a significant proportion of obese individuals in the general population can exhibit a phenotype free of metabolic abnormalities [6, 9, 10]. Consequently, recent studies have suggested that the differentiation of metabolic health status among obese individuals is partially due to different fat distribution [38]. Abdominal visceral fat accumulation or central obesity is currently known to be the key risk factor that links to metabolic abnormalities in obesity [6, 10, 11]. Essentially, centrally obese patients have significantly higher risk of one or more abnormalities related to lipids, insulin, glucose, blood pressure and inflammation than peripherally obese patients [4, 5, 8]. However, the specific downstream metabolic characteristic and molecular mechanisms which distinguish the metabolically unhealthy obese phenotype from metabolically healthy obese phenotype remain poorly understood.

The criteria applied to define metabolically unhealthy and healthy obesity vary largely in the current literature [6–12]. Apparently, the stringency of criteria used in a study will affect the numbers and types metabolites that will be detected. To date, three studies have been performed using metabolomics technology to address the metabolic profiles between metabolically unhealthy and healthy obese individuals [20–22]. However, none of the studies considered the role of fat distribution and neither had normal weight control group. In our current study, on top of defining different phenotypes of obesity with waist circumference, which is known as an effective predictor abdominal visceral fat accumulation [39, 40], stricter criteria were taken with both MS and IR been used when defining metabolically healthy and unhealthy obesity [9, 11]. Moreover, visceral fat mass and visceral fat percentage were measured and found significantly higher in MUCO group. Furthermore, the aforementioned groups were matched for age, sex, total dietary calorie intake, physical activity and BMI to eliminate the confounding effects of these factors on metabolites. Age and sex are primarily important factors that affect metabolism and the metabolites studied [41–43]. Total calorie intake is a critical factor in maintaining energy balance and likely the levels of various metabolites: amino acids, lipids and carbohydrates [44]. In addition, physical activity level is likely the most important non-dietary factor to influence the general metabolism of all the macro- and micro-nutrients [44, 45]. It is extremely important to properly control these factors in a study that analyzes and compares hundreds of metabolites. Moreover, a NW group was added as the control group in the validation stage to further emphasize differences in obesity associated risk factors related to metabolic health. These stringency are important and indicate our findings are likely more specific and accurate.

In the validation stage, we successfully confirmed the elevation of 5 serum metabolites (leucine, isoleucine, valine, alphaAAA, C3 acylcarnitine) discovered in the first stage that are associated with metabolically unhealthy centrally obese patients. No significant difference was found between MHPO and NW control group. Leucine, isolucine, and valine are BCAAs. BCAAs comprise approximately 40 % of the free essential amino acids in blood. They play important roles in maintenance and growth of skeletal muscle, are used as an energy source during exercise, and can serve as gluconeogenic precursors [46]. In humans, serum BCAAs cannot be created from other compounds and mainly come from dietary protein, amino acids and endogenous protein catabolism in muscle [47]. The two groups of obese patients from our study had no significant difference in dietary intake of protein or amino acids (shown in Additional file 2: Table S2 and Additional file 3: Table S3). These results suggest that the significantly higher level of BCAAs in MUCO individuals may be due to the increased protein catabolism, likely secondary to insulin resistance [14, 48]. A recent theory suggested that increased circulating concentration of BCAAs might be caused by a block of BCAAs catabolism in the mitochondria of adipose tissue [48–51], with visceral fat playing an important role in this regard [52]. Furthermore, accumulating evidence indicated that abnormal levels of circulating BCAAs were involved in various diseases, including chronic liver disease [53], obesity [54], diabetes [55], cardiovascular disease [56] and cancer [57]. It has long been recognized that BCAAs are elevated in the blood of patients with obesity, IR or diabetes [14, 54, 55, 58–60]. The finding from our present study provides the first link between increased serum BCAAs levels with central obesity specifically, rather than with peripheral obesity. The precise molecular mechanisms mediating the association between BCAAs and metabolic abnormalities during obesity is unclear, but may be related to the activation of mTOR-S6 K1 induced disruption of insulin signaling or the inhibition of GCN2, ATF4 and AMPK mediated lipid, glucose metabolism and energy homeostasis disorder [14, 57, 61].

Acylcarnitines are formed intracellularly from carnitine during the metabolism of long-chain fatty acids and BCAAs [62, 63]. In our study, we discovered that serum acylcarnitine C3 (propionylcarnitine) were significantly higher in MUCO individuals. C3 is a product of BCAAs mitochondrial catabolism, especially isoleucine and valine catabolism [14]. Elevation of serum C3 levels was also observed in patients with obesity or diabetes previously [14, 64–66], and the increased BCAA degradation in muscle tissue or liver associated with the increased serum BCAA levels was considered as a potential cause [14].

Biogenic amines are bioactive substances containing one or more amine groups. They are basic nitrogenous compounds formed mainly by decarboxylation of amino acids or by amination and transamination of aldehydes and ketones. Among the biogenic amines measured in the current study, only serum alpha-aminoadipic acid (alphaAAA) was significantly different among the groups. AlphaAAA is a product of lysine degradation in mammals [67, 68], that has been identified as a biomarker for the development of T2DM and a potential modulator of glucose homeostasis in humans [69, 70]. Reports on the changes of serum alphaAAA level in obesity are rare, while a study from Korea described significantly higher serum alphaAAA levels in obese children [71]. It is well known that obesity, especially central obesity is the primary risk factor for the development of IR and diabetes [6, 10–12]. However the factors that link obesity and diabetes are largely unknown. The patients in the present study were well defined having central obesity. Our finding strongly suggests that alphaAAA is at least one important factor mediating central obesity and diabetes.

This study had a number of limitations to consider. First, this is a cross-sectional case–control study. Sequential observations made in a prospective manner would provide more useful information. Secondly, although the targeted metabolomics approach explored 186 metabolites, we might have missed important metabolites which the panel does not have. Finally, although HOMA-IR is a widely accepted measure of IR, hyperinsulinemic euglycemic clamp technique is considered a more accurate method to measure IR [72].

## Conclusions

This is the first study using a targeted metabolomics approach and two-stage study design to identify serum metabolites differences between metabolically healthy peripheral obese and unhealthy central obese individuals. We found significantly higher levels of serum branched-chain amino acids (leucine, isoleucine, valine), propionylcarnitine (C3 acylcarnitine) and alphaAAA to distinguish metabolically unhealthy central obesity from metabolically healthy peripheral obesity. The identified metabolites provide novel insights into the metabolic characteristic and pathogenesis of metabolic abnormalities in central obesity. Future studies are warranted to further verify the relevance of these novel metabolites associated with central obesity, and elucidate the underlying biochemical mechanisms.

## Additional files

Additional file 1: Table S1. List of metabolite concentrations determined using the Biocrates AbsoluteIDQ kit. (DOC 42 kb) Additional file 2: Table S2. Dietary amino acids intakes of the study participants in the discovery stage. (DOC 46 kb) Additional file 3: Table S3. Dietary amino acids intakes of the study participants in the validation stage. (DOC 51 kb)

## Abbreviations

AF%: percent android fat
BCAA: branched-chain amino acids
BF%: total percent fat
BMI: body mass index
BP: blood pressure
CVD: cardiovascular disease
DBP: diastolic blood pressure
DXA: dual-energy X-ray absorptiometry
FFQ: food frequency questionnaire
GF%: percent gynoid fat
HDL-C: high-density lipoprotein cholesterol
HOMA-IR: homeostasis model assessment of insulin resistance
IR: insulin resistance
lysoPC: lysophosphatidylcholine
MAO: metabolic abnormal obesity
MHO: metabolic healthy obesity
MHPO: metabolically healthy peripherally obesity
MS: metabolic syndrome
MUCO: metabolically unhealthy centrally obesity
NW: normal weight
PC: phosphatidylcholines
SBP: systolic blood pressure
SM: sphingomyelin
T2DM: type 2 diabetes mellitus
TF%: percent trunk fat
TG: triglycerides
VF%: percent visceral fat
