# Supplementary material for: Serum metabolic biomarkers distinguish metabolically healthy peripherally obese from unhealthy centrally obese individuals
Source: Nutr Metab (Lond). 2016 May 12;13:33. doi: 10.1186/s12986-016-0095-9 (PMC4865032; doi:10.1186/s12986-016-0095-9)
Supplement: Additional file 1: Table S1. — List of metabolite concentrations determined using the Biocrates AbsoluteIDQ kit. (DOC 42 kb) [file 12986_2016_95_MOESM1_ESM.doc]

**Supplementary Table 1** List of metabolite concentrations determined using the Biocrates AbsoluteIDQ kit

| Metabolite class | Number | Metabolite name or abbreviation | Biological relevance (selected examples) |
| --- | --- | --- | --- |
| Amino acids | 21 | Alanine, arginine, asparagine, aspartate, citrulline, glutamine, glutamate, glycine, histidine, isoleucine, leucine, lysine, methionine, ornithine, phenylalanine, proline, serine, threonine, tryptophan, tyrosine, valine | Amino acid metabolism, urea-cycle, activity of  gluconeogenesis and glycolysis, insulin  sensitivity, neurotransmitter metabolism, oxidative stress |
| Acylcarnitine | 40 | C0, C2,C3 ,C3:1, C3OH, C4, C41, C3DCC4OH, C5, C51, C51DC, C5DCC6OH, C5MDC, C5OHC3DCM, C6C41DC, C61, C7DC, C8, C9, C10, C101, C102, C12, C121, C12DC, C14, C141, C141OH, C142, C142OH, C16, C161, C161OH, C162, C162OH, C16OH, C18, C181, C181OH, C182 | Energy metabolism, fatty acid transport and  mitochondrial fatty acid oxidation, ketosis,  oxidative stress, mitochondrial membrane  damage |
| Biogenic amines | 12 | Asymmetric dimethylarginine, total dimethylarginine, alpha-Aminoadipic acid ,creatinine, kynurenine, Methioninesulfoxide, putrescine, sarcosine, serotonin, spermidine, spermine taurine | Neurological disorders, cell proliferation, cell cycle progression, DNA stability, oxidative stress |
| Lysophosphatidylcholines | 14 | lysoPCaC14:0/C16:0/C16:1/C17:0/C18:0/C18:1/C18:2/C20:3/C20:4/C26:0/C26:1/C28:0/C28:1 | Degradation of phospholipids, membrane  damage, signalling cascades, fatty acid profile |
| Diacyl-phosphatidylcholines | 37 | PC aa C24:0/C26:0/C28:1/C30:0/ C32:0/C32:1 / C32:2/C32:3/C34:1/C34:2/C34:3/C34:4/C36:0/C36:1/C36:2/C36:3/C36:4/C36:5/C36:6/C38:0/C38:1/C38:3/C38:4/C38:5/C38:6/C40:1/C40:2/C4  0:3/C40:4/C40:5/C40:6/C42:0/C42:1/C42:2/C42:4/C42:5/C42:6 | Dyslipidaemia, membrane composition anddamage, fatty acid profi le, activity of  Acyl-alkyl- desaturases |
| Acyl-alkyl-  phosphatidylcholines | 38 | PCaeC30:0/C30:1/C30:2/C32:1/C32:2/C34:0/C34:1/C34:2/C34:3/C36:0/C36:1/C36:2/C36:3/C36:4/C36:5/C38:0/C38:1/C38:2/C38:3/C38:4/C38:5/C38:6/C40:1/C40:2/C40:3/C40:4/C40:5/C40:6/C42:0/C42:1/C42:2/C42:3/C42:4/C42:5/C44:3/C44:4/C44:5/C44:6 |
| Sphingomyelins | 15 | SM C16:0, SM C16:1, SM C18:0, SM C18:1, SM C20:2, SM C22:3, SM C24:0, SM 24:1, SM C 26:0, SM C 26:1, SM (OH) C14:1, SM (OH) C16:1, SM (OH) C22:1, SM (OH) C22:2,SM (OH) C24:1 | Signalling cascades, membrane damage (eg,  neurodegeneration) |
| Hexose | 1 | H1 | Carbohydrate metabolism |
| Total | 178 |  |  |

aa, acyl-acyl; ae, acyl-alkyl; a, lyso; Cx:y, where x is the number of carbons in the fatty acid side chain; y is the number of double bonds in the fatty acid side chain; DC, decarboxyl; M, methyl; OH, hydroxyl; PC, phophatidylcholine; SM, sphingomyelin.
