# Supplementary material for: Serum metabolic biomarkers distinguish metabolically healthy peripherally obese from unhealthy centrally obese individuals
Source: Nutr Metab (Lond). 2016 May 12;13:33. doi: 10.1186/s12986-016-0095-9 (PMC4865032; doi:10.1186/s12986-016-0095-9)
Supplement: Additional file 2: Table S2. — Dietary amino acids intakes of the study participants in the discovery stage. (DOC 46 kb) [file 12986_2016_95_MOESM2_ESM.doc]

**Supplementary Table 2 Dietary amino acids intakes of the study participants in the discovery stage**

| **Variables** | **MUCO** | **MHPO** | ***P*** |
| --- | --- | --- | --- |
| Histidine(g) | 2.36 ± 2.22 | 1.89 ± 0.66 | 0.558 |
| Isoleucine(g) | 4.26 ± 4.67 | 3.15 ± 1.10 | 0.496 |
| Leucine(g) | 7.30 ± 7.64 | 5.55 ± 1.83 | 0.513 |
| Lysine(g) | 6.27 ± 6.36 | 4.81 ± 1.80 | 0.518 |
| Methionine(g) | 1.9 ± 2.02 | 1.51 ± 0.51 | 0.515 |
| Phenylalanine(g) | 3.88 ± 3.82 | 3.01 ± 0.96 | 0.520 |
| Threonine(g) | 3.39 ± 3.58 | 2.56 ± 0.87 | 0.512 |
| Tryptophan(g) | 1.01 ± 1.07 | 0.75 ± 0.25 | 0.493 |
| Valine(g) | 4.91 ± 5.23 | 3.69 ± 1.26 | 0.506 |
| Alanine(g) | 3.74 ± 3.10 | 3.05 ± 0.97 | 0.531 |
| Arginine(g) | 4.13 ± 3.30 | 3.37 ± 1.11 | 0.525 |
| Aspartic acid(g) | 7.55 ± 6.31 | 5.76 ± 1.94 | 0.427 |
| Cystine(g) | 1.10 ± 0.78 | 0.87 ± 0.22 | 0.419 |
| Glutamic acid(g) | 16.62 ± 16.34 | 12.93 ± 4.05 | 0.521 |
| Glycine(g) | 2.86 ± 2.12 | 2.38 ± 0.80 | 0.551 |
| Hydroxyproline(g) | 0.061 ± 0.051 | 0.031 ± 0.023 | 0.167 |
| Proline(g) | 6.49 ± 7.26 | 4.76 ± 1.75 | 0.497 |
| Serine(g) | 4.15 ± 4.24 | 3.15 ± 1.00 | 0.498 |
| Tyrosine(g) | 3.29 ± 3.70 | 2.45 ± 0.87 | 0.517 |

All values are means ± SDs.The independent *t*-test was set to p < 0.05.
