# Supplementary material for: Serum metabolic biomarkers distinguish metabolically healthy peripherally obese from unhealthy centrally obese individuals
Source: Nutr Metab (Lond). 2016 May 12;13:33. doi: 10.1186/s12986-016-0095-9 (PMC4865032; doi:10.1186/s12986-016-0095-9)
Supplement: Additional file 3: Table S3. — Dietary amino acids intakes of the study participants in the validation stage. (DOC 51 kb) [file 12986_2016_95_MOESM3_ESM.doc]

**Supplementary Table 3** Dietary amino acids intakes of the study participants in the validation stage

| Variables | NW | MUCO | MHPO | *P1* | *P1* | *P3* |
| --- | --- | --- | --- | --- | --- | --- |
| Histidine(g) | 1.83±1.19 | 1.52±0.98 | 2.08 ±0.97 | 0.571 | 0.681 | 0.116 |
| Isoleucine(g) | 3.06±2.01 | 2.54±`1.73 | 3.45 ±1.67 | 0.602 | 0.727 | 0.151 |
| Leucine(g) | 5.35±3.51 | 4.43 ±2.83 | 6.04±2.82 | 0.565 | 0.705 | 0.123 |
| Lysine(g) | 4.69±3.16 | 3.89 ± 2.64 | 5.37 ± 2.56 | 0.600 | 0.673 | 0.124 |
| Methionine(g) | 1.45±0.88 | 1.22±0.80 | 1.66 ±0.77 | 0.614 | 0.639 | 0.116 |
| Phenylalanine(g) | 2.98±1.95 | 2.46 ±1.55 | 3.30±1.54 | 0541 | 0.783 | 0.150 |
| Threonine(g) | 2.58±1.67 | 2.14 ±1.50 | 2.91 ±1.42 | 0.600 | 0.732 | 0.153 |
| Tryptophan(g) | 0.75±0.48 | 0.63 ± 0.41 | 0.84 ±0.40 | 0.601 | 0.758 | 0.168 |
| Valine(g) | 3.57±2.32 | 2.98 ± 1.94 | 4.02 ± 1.90 | 0.585 | 0.729 | 0.143 |
| Alanine(g) | 3.15 ±2.02 | 2.60 ± 1.61 | 3.55 ± 1.64 | 0.539 | 0.771 | 0.114 |
| Arginine(g) | 3.63±2.37 | 2.97 ±1.82 | 4.03±1.99 | 0.524 | 0.778 | 0.138 |
| Aspartic acid(g) | 6.19±3.91 | 5.30±3.45 | 6.83±3.17 | 0.666 | 0.803 | 0.240 |
| Cystine(g) | 0.92± 0.67 | 0.75±0.35 | 1.00±0.41 | 0.419 | 0.846 | 0.123 |
| Glutamic acid(g) | 12.66±7.90 | 11.03±8.11 | 14.06±6.50 | 0.745 | 0.796 | 0.296 |
| Glycine(g) | 2.57±1.69 | 1.13±1.29 | 2.88±1.40 | 0.552 | 0.743 | 0.135 |
| Hydroxyproline(g) | 0.04±0.04 | 0.03± 0.03 | 0.04 ± 0.03 | 0.791 | 0.999 | 0.782 |
| Proline(g) | 4.38±2.77 | 3.71±2.24 | 4.86±2.34 | 0.629 | 0.779 | 0.198 |
| Serine(g) | 3.11±2.06 | 2.57±1.65 | 3.42±1.59 | 0.557 | 0.815 | 0.178 |
| Tyrosine(g) | 2.30±1.52 | 1.93±1.34 | 2.62±1.25 | 0.632 | 0.708 | 0.157 |

All values are means ± SDs. The One-Way ANCOVA followed  by *Tukey* test was set to p<0.05.

*P1*:The P value between MUCO and NW groups; *P2*:The P value between MHPO and NW groups; *P3*:The P value between MUCO and MHPO groups
